# Effect of arotinolol on chronic heart failure: A systematic review and meta-analysis of randomized controlled trials

**DOI:** 10.3389/fcvm.2022.1071387

**Published:** 2022-12-14

**Authors:** Pingping Huang, Qingya Song, Yifei Wang, Anzhu Wang, Lijun Guo, Hongwei Zhang, Zhibo Zhang, Xiaochang Ma

**Affiliations:** ^1^Xiyuan Hospital, China Academy of Chinese Medical Sciences, Beijing, China; ^2^Graduate School, China Academy of Chinese Medical Sciences, Beijing, China; ^3^Xiyuan Hospital, Beijing University of Chinese Medicine, Beijing, China; ^4^National Clinical Research Center for Chinese Medicine Cardiology, Beijing, China

**Keywords:** arotinolol, heart failure, cardiac insufficiency, randomized controlled trial, systematic evaluation, meta-analysis

## Abstract

**Background:**

Heart failure is the end stage of all cardiovascular diseases, which brings a heavy burden to the global health network. Arotinolol, as a new type of β Receptor blocker, has a good antihypertensive effect. Many clinical trials have observed the clinical efficacy of arotinolol in the treatment of essential hypertension. However, so far, there has been no systematic evaluation on the efficacy and safety of arotinolol in the treatment of chronic heart failure.

**Objective:**

The purpose of this review was to systematically evaluate the clinical efficacy of arotinolol in patients with chronic heart failure.

**Methods:**

Randomized controlled trials (RCTs) of arotinolol in the treatment of chronic heart failure were retrieved from seven databases according to the Cochrane manual, including CNKI (China National Knowledge Infrastructure), Wan fang database, VIP database, PubMed, Sinomed, EMBASE, and the Cochrane Library databases. The main outcomes were the effective rate, left ventricular ejection fraction (LVEF), blood pressure, heart rate, cardiac index, stroke volume (SV), brain natriuretic peptide (BNP), hypersensitive C-reactive protein (Hs-CRP), left ventricular end diastolic volume (LVEDV), left ventricular end diastolic diameter (LVEDD), and adverse events (AEs).

**Results:**

A total of 17 trials met the qualification criteria, which included 1,717 patients with heart failure. Most trials had uncertain risks in terms of random sequence generation, allocation hiding, patient loss, and result evaluation. Meta analysis showed that arotinolol significantly improved the treatment efficiency of patients with heart failure (standardized mean difference (SMD) = 4.07, 95% confidence interval (CI) [2.89, 5.72], *p* = 0.00, *I*^2^ = 0), LVEF (SMD = 1.59, 95% CI [0.99, 2.19], *p* = 0.000 0, *I*^2^ = 95.8%), cardiac index (SMD = 0.32, 95% CI [0.11, 0.53], *p* = 0.03), *I*^2^ = 0), SV (SMD = 2.00, 95% CI [1.57, 2.34], *p* = 0.000, *I*^2^ = 64.2%), lower BNP (SMD = −0.804, 95% CI [−0.97, −0.64], *p* = 0.000, *I*^2^ = 94.4%), and LVEDV (SMD = −0.25, 95% CI [−0.45, −0.05], *p* = 0.015, *I*^2^ = 0). There was no statistical significance for blood pressure (SMD_systolic pressure_ = −0.09, 95% CI [−0.69, 0.51], *p* = 0.775, *I*^2^_systolic pressure_ = 90.2%; SMD_diastolic pressure_ = −0.16, 95% CI [−0.79, 0.48], *P* = 0.632, *I*^2^_diastolic pressure_ = 91.2%), heart rate (SMD = −0.12, 95% CI [−1.00, 0.75], *P* = 0.787, *I*^2^ = 96.1%), Hs-CRP (SMD = −1.52, 95% CI [−3.43, 0.40], *P* = 0.121, *I*^2^ = 98.3%), and LVEDD (SMD = −0.07, 95% CI [−0.90, 0.76], *P* = 0.870, *I*^2^ = 96.5%).

**Conclusion:**

Arotinolol can safely and effectively improve the effective rate of patients with chronic heart failure, increase LVEF, increase CI and SV, and reduce BNP and LVEDV. However, because of the low overall quality of the included randomized controlled trials, these findings need to be considered carefully. More high-quality randomized controlled trials are needed for further verification, to provide a more scientific basis for the safety and effectiveness of arotinolol in the clinical treatment of heart failure.

**Systematic review registration:**

[https://www.crd.york.ac.uk/PROSPERO/display_record.php?RecordID=371214], identifier [CRD:420223371214].

## Introduction

Chronic heart failure (CHF) is a disorder in which the systolic and/or diastolic function of the heart becomes impaired because of myocardial strain and decreases in the ejection fraction, eventually leading to blood pooling in the venous system and insufficient perfusion in the arterial system (1). The new guidelines (2) classified heart failure according to left ventricle ejection fraction (LVEF), LVEF ≤40% is defined as heart failure with reduced ejection fraction (HFrEF), LVEF between 41 and 49% is called heart failure with mildly reduced ejection fraction (HFmrEF), when LVEF ≥50%, this type of heart failure is defined as heart failure with preserved ejection fraction (HFpEF). In addition, previous LVEF ≤ 40% and a follow-up measurement of LVEF >40% is referred to heart failure with improved ejection fraction (HFimpEF). In the early stage of HFrEF disease, activation of the sympathetic nervous system and the renin-angiotensin-aldosterone system (RAAS) can compensate for cardiac injury, but prolonged activation of these pathways can lead to deterioration of cardiac function (3). 2021 ESC Guidelines (1) changed the standard “Golden Triangle” protocol to the “New Quadruplex” standard treatment protocol, which consisted mainly of angiotensin-converting enzyme inhibitors (ACEI) or angiotensin II receptor blockers (ARB) or angiotensin receptor enkephalase inhibitors (ARNI), beta receptor blockers, mineralocorticoid receptor antagonists (MRA), and sodium glucose co-transporters 2 inhibitors (SGLT2i). Additionally, Ivabradine, a soluble guanyl cyclase agonist, reduced the length of hospitalization for heart failure in patients at high risk for HFrEF (4). Hydralazine/Isosorbide Dinitrate caused vasodilation by enhancing nitric oxide signaling and improved the prognosis of patients with HFrEF (5). There are no clinical trials conducted specifically in patients with HFmrEF, and the current evidence came from subgroup analyses of clinical trials with patients with HFpEF and HFrEF as study populations. These studies (6–8) suggested that the use of conventional therapeutic agents for HFrEF in a population of patients with ejection fraction of 40–50% can be meaningful in terms of reducing mortality, readmission rates, and other endpoint events. HFpEF accounts for more than half of heart failure cases, and diastolic dysfunction is an essential component of the pathophysiological basis of HFpEF, but multiple cardiac and vascular factors and non-cardiac abnormalities are also involved (9). Although cardiovascular mortality is lower in the HFpEF group than in the HFrEF group, there is a high frequency of rehospitalization and a poor quality of life (10). In addition, there is no clear effective treatment for HFpEF in clinical trials (11). In the 2019 Heart Failure Association (HFA) ATLAS program, 13 European countries reported data showing a median heart failure prevalence estimate of 17 per 1,000 people, ranging from <12 in Greece and Spain to >30 per 1,000 people in Lithuania and Germany (12). The 2021 American Heart Association statistical update estimated the prevalence of heart failure at 6 million people, or 1.8% of the total US population (13). CHF has been defined as a global pandemic, and its prevalence is expected to increase due to improved survival after diagnosis of heart failure with the availability of life-saving evidence-based treatment and increased overall life expectancy in the general population (14). The burden of CHF on health care expenditures worldwide is worrisome in 2012, the total cost of CHF in the United States was estimated at $30.7 billion, and projections indicate that by 2030, the cost will increase 127% to $69.8 billion (15), equivalent to approximately $244 per U.S. adult. These disturbing trends reflect the complexity of CHF syndromes.

The use of β receptor blockers is the main means of drug treatment for patients with CHF. In HFrEF, the use of β receptor blockers use has been proven to reduce death, cardiovascular related death and sudden cardiac death (11), and related clinical studies (16) have also proved their effectiveness in reducing the hospitalization rate of heart failure. Carvedilol, metoprolol and bisoprolol have the strongest evidence in HFrEF, based on their benefit in reducing mortality in large RCT (17–20). In addition, carvedilol also inhibits α_1_ receptor, which may have additional therapeutic value, as observed in COMET RCT (21) the use of carvedilol can reduce the death rate by 20% compared with metoprolol in HFrEF population.

Arotinolol is a new receptor inhibitor similar to carvedilol, which can block both α and β receptors to exert a strong inhibitory effect on sympathetic tone, and can effectively lower the heart rate and reduce the excitability of the sympathetic nervous system (22). Arotinolol is often used in clinical practice as a therapeutic agent for patients with hypertension. A multi-center clinical study enrolling patients with dipper and non-dipper hypertension who were given arotinolol (40 mg per day) for 4 weeks showed that arotinolol lowered nighttime blood pressure levels more significantly in patients with non-dipper hypertension, helping to restore the circadian rhythm of blood pressure (22). Arotinolol can also be used to treat essential tremor, and the results of a randomized crossover trial showed that taking arotinolol 20 mg twice a day is more effective than taking propranolol 80 mg twice a day in treating essential tremor (23). In addition, a study showed that (24) patients with CHF are often associated with increased sympathetic excitability and elevated levels of catecholamines, renin, and angiotensin in their circulating blood. Arotinolol reduces coronary artery resistance and dilates coronary arteries through α receptor blocking effects. Through β-blockade, it inhibits cardiac hyperfunction, reduces myocardial oxygen consumption, and counteracts the over-activation of sympathetic nervous system, neurohormones and the renin–angiotensin system (RAS) system, thus improving cardiac function. It is more advantageous in the treatment of cardiac diseases compared with β-blockers alone. A clinical trial (25) showed that arotinolol alone also significantly improves left heart function in patients with CHF, resulting in a significant decrease in plasma BNP levels, and is well tolerated. In this study, we investigated the therapeutic effects of arotinolol in patients with CHF using a systematic evaluation approach.

## Methods

### Data sources and searches

We performed a systematic literature search of CNKI (China National Knowledge Infrastructure), the Wan-fang database, the VIP database, PubMed, Sinomed, EMBASE, and the Cochrane Library databases, as well as meeting minutes and clinical trial databases of ongoing and unpublished trials. A reference list of all articles obtained through the search process was reviewed to identify further relevant studies. Specifically, the keywords utilized for this search were: (“heart failure” or “cardiac decompensation”) and (“arotinolol” or “arotinolol hydrochloride”). As of August 2022, the search scope was limited to articles about humans.

### Inclusion and exclusion criteria

#### Types of studies

We included randomized controlled trials (RCTs).

#### Types of participants

Patients were diagnosed with CHF according to any accepted criteria. There were no restrictions in terms of age, sex and duration of disease.

#### Types of interventions

Intervention groups were treated with arotinolol plus conventional treatments (CTs), while control groups were treated with the CTs. Refer to the 2022 AHA/ACC/HFSA guidelines for the management of heart failure (2). CTs include angiotensin-converting enzyme inhibitor (ACEI), angiotensin receptor blockers (ARB), angiotensin receptor/enkephalase inhibitor (ARNI), β receptor blockers, aldosterone receptor antagonists (ARB), sodium glucose co-transporter 2 inhibitors (SGLT2i), lipid lowering drugs, diuretics, hydralazine/isosorbide dinitrate, digoxin and other drugs for primary cardiovascular disease.

#### Types of outcomes

Effective rate, left ventricular ejection fraction (LVEF), blood pressure, heart rate, cardiac index, stroke volume (SV), brain natriuretic peptide (BNP), hypersensitive C-reactive protein (Hs-CRP), left ventricular end diastolic volume (LVEDV), left ventricular end diastolic diameter (LVEDD), and adverse events (AEs).

Studies were excluded because of: (1) Incomplete data; (2) duplicate publications (3) case reports, reviews, animal studies, conference abstracts, letters, and expert opinions.

### Data extraction and quality assessment

Two researchers (Pingping Huang and Zhibo Zhang) independently retrieved the first author’s name, year of publication, study duration, sample size, interventions, results, and adverse events from the included literature. Using the Cochrane Handbook for the Systematic Evaluation of Interventions, the methodological quality of each included randomized controlled trial was evaluated [Higgins (26)]. Seven characteristics were used, including sequence generation, allocation concealment, participant and staff masking, outcome assessor masking, incomplete outcome data, selective result reporting, and other sources of bias. A graph was made to show the results of the evaluation of each item’s quality using the three levels of bias: “low risk,” “high risk,” or “unclear danger.” When the analysis covered more than ten articles, the funnel lots were used to assess publication bias.

### Statistical analysis

Stata 14.0 (StataCorp LLC, College Station, TX, USA) and Revman 5.3 (Cochrane, London, UK) were used to do the entire meta-analysis. Effect estimates, including objective response (OR), response rate (RR) and raw data, were extracted and represented as standardized mean difference (SMD) or OR and 95% CI for statistical analysis. Statistical tests Q (qualitative) and *I*^2^ were used to evaluate heterogeneity (quantitative). The outcomes of the heterogeneity test in each study guided the choice of the statistical model. A fixed effects model was used when *P* ≥ 0.1 and *I*^2^ <50%, indicating no or low heterogeneity between the literature. When *P* < 0.1 and *I*^2^ > 50%, on the other hand, this indicated significant heterogeneity in the study data and the random effects model was used in this situation. *I*^2^ was utilized as the primary evaluation when the two heterogeneity tests produced conflicting results. To explain the source of heterogeneity, sensitivity analysis, subgroup analysis, and meta regression were used.

## Results

### Description of included trials

**FIGURE 1:**
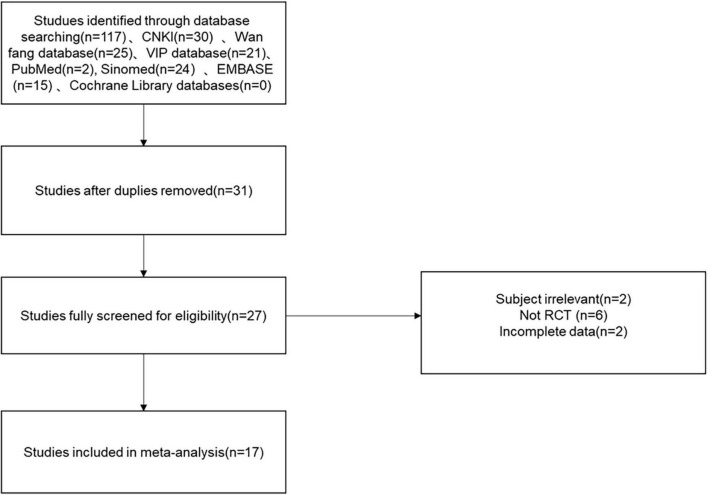
Flow chart of the study selection process showing how we screened eligible randomized controlled trials.

A total of 117 relevant studies were initially examined in the databases, including CNKI: 30, Wan fang database: 25, VIP: 21, Sinomed: 24, Embase: 15, Pubmed: 2. Next, 86 studies were excluded, 4 studies were excluded from the initial screening by reading titles and abstracts, the remaining 27 were potentially included in the study, and 10 were then excluded by downloading the full-text and re-screening. Finally, the quantitative synthetic Meta-analysis included 17 papers with a total of 1,717 patients (27–43), comprising 860 patients in the trial group and 857 patients in the control group, all of which were randomized controlled trials. The data filtering process can be viewed in Figure 1. Thirteen trials (27–32, 34, 35, 37–40, 42) reported the overall effective rate; fourteen (28–31, 33–37, 39–43) reported the left ventricular ejection fraction (LVEF); six (32, 33, 35, 36, 39, 41) reported blood pressure; seven (32–36, 39, 41) reported heart rate; three reported (34, 37, 42) the cardiac index and stroke volume (SV); six (27, 30, 35, 39–41) reported BNP; four (31, 36, 40, 42) reported hypersensitive C-reactive protein (Hs-CRP); four (29, 35, 40, 41) reported left ventricular end diastolic volume (LVEDV); and eight (28, 31, 33–37, 42) reported left ventricular end diastolic diameter (LVEDD). The basic characteristics of the seventeen included RCTs are described in Table 1. All of the seventeen included RCTs (27–43) mentioned “randomization,” six (27, 29, 32, 37, 40, 42) described specific randomization methods; however, no studies mentioned whether they were blinded, two articles (36, 39) had incomplete data, and all studies had a low risk of selective reporting bias. All included studies were comparable at baseline. The methodological quality assessment and characteristics of the included studies can be detailed in Supplementary Figure 1 and Table 1 in Supplementary material.

**TABLE 1:** Subgroup analysis of the included studies.

Outcome	Treatment duration	*n*	*P*	*I* ^2^	Random-effects model HR (95% CI)
Duration	Less than 4 months	8	0.000	96.3	1.75 [0.88, 2.62]
	More than 4 months	8	0.000	93.9	1.32 [0.7, 1.93]
Dosage	Daily dosage ≤ 10 mg/d	2	0.000	96.7	1.71 [0.81, 2.6]
	10 mg/d < daily dosage ≤ 20 mg/d	8	0.007	86.2	1.29 [0.6, 1.99]
	Daily dosage > 20 mg/d	6	0.000	94.3	1.39 [0.57, 2.2]
Age	• Less than 60 years old	8	0.000	96.7	1.57 [0.67, 2.08]
	• More than 60 years old	8	0.000	93.1	1.49 [0.9, 2.48]

HR, hazard ratio; CI, confidecne interval.

### Effective rate

**FIGURE 2:**
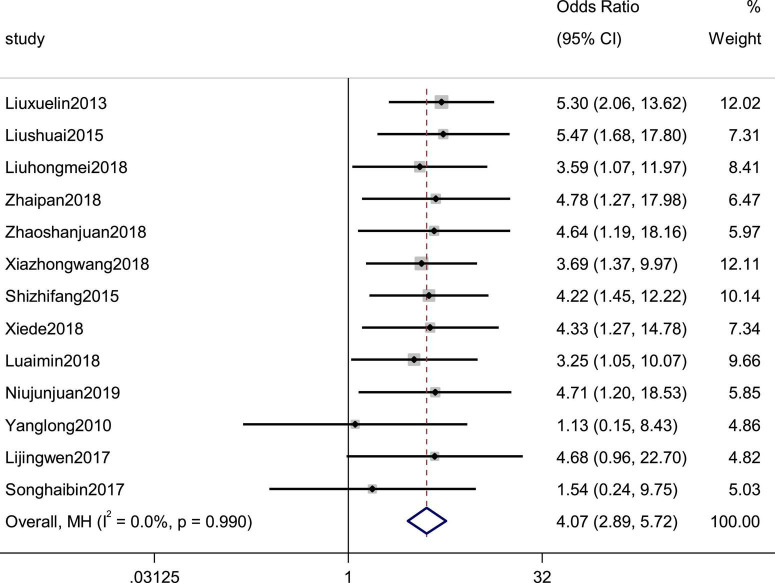
Forest plot of the effective rate in patients with heart failure treated with conventional therapy plus arotinolol (experimental) or conventional therapy alone (control). Weights are from Mantel-Haenszel model.

**FIGURE 3:**
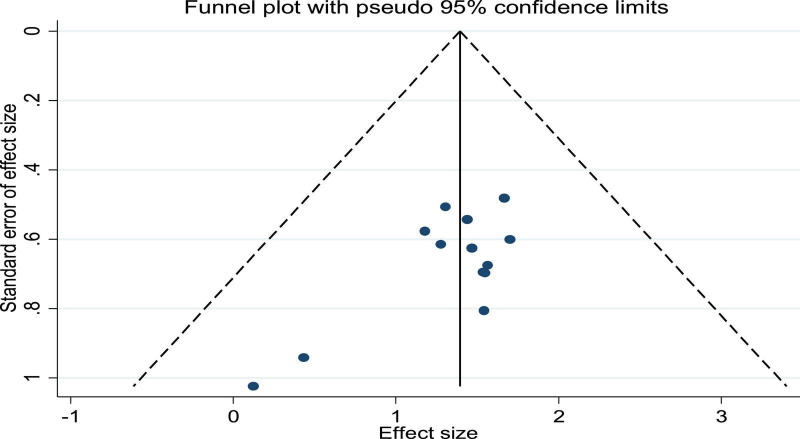
Funnel plot of the publication bias for RCTs of patients with heart failure treated with conventional therapy plus arotinolol (experimental) or conventional therapy alone (control).

Thirteen studies reported efficacy as an outcome. There was little heterogeneity in the study (*p* = 0.99, *I*^2^ = 0), which indicated that the treatment group was significantly superior to the control group in improving the efficiency of symptoms in patients with CHF, using a fixed-effects model to combine the effect sizes (SMD = 4.07, 95% CI [2.89, 5.72], *p* = 0.00; Figure 2). There was also no evidence of publication bias based on funnel plot inspection (Figure 3).

### Left ventricular ejection fraction

**FIGURE 4:**
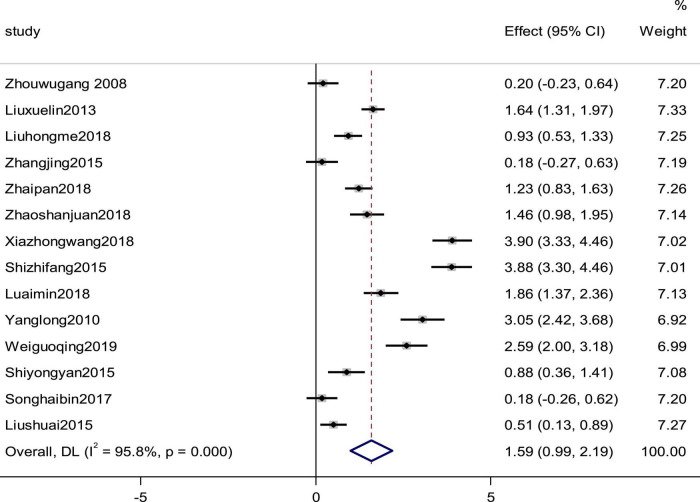
Forest plot of the LVEF in patients with heart failure treated with conventional therapy plus arotinolol (experimental) or conventional therapy alone (control). Weights are from random-effects model.

LVEF was reported by 14 studies as an observation index. High heterogeneity (*p* = 0.000, *I*^2^ = 95.8%), prompted us to use a random effects model to analyze the data. The meta-analysis results indicated that the combination of western medicine and arotinolol could further increase the LVEF compared with western medicine alone (SMD = 1.59, 95% CI [0.99, 2.19], *p* = 0.0000 (Figure 4). In addition, sensitivity analyses were conducted, removing one study at a time and analyzing the other studies to estimate whether the results were significantly influenced by a single study. The sensitivity analyses showed that the combined effect sizes were similar and the results were robust (Supplementary Figure 2). In fact, research characteristics such as the course of the disease and basic diseases might also lead to heterogeneity. However, some studies did not provide these baseline data completely; therefore, we could not conduct meta regression on these factors. Finally we used subgroup analysis and meta regression on other factors, which indicated that the improvement in the LVEF summarized value did not depend on the duration, daily dosage, and age (Tables 1, 2).

**TABLE 2:** Meta-regression of the included studies.

Heterogeneity factor	Coefficient	Standard error (SE)	*t*	*p*-value	95% confidence interval (CI)
Duration	–0.420557	0.6189065	–0.68	0.508	−1.747979, 0.9068654
Age	–0.2810217	0.4884985	–0.58	0.574	−1.328747, 0.7667033
Dosage	–0.0492256	0.474339	–0.10	0.919	−1.066582, 0.9681304
Sample size	0.652401	0.6089736	1.07	0.302	−0.6537176, 1.95852

### Systolic pressure

**FIGURE 5:**
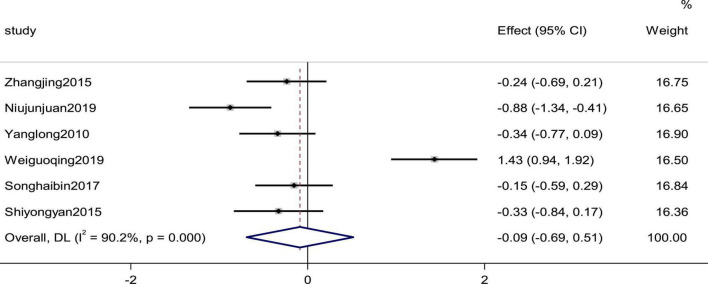
Forest plot of the systolic pressure in patients with heart failure treated with conventional therapy plus arotinolol (experimental) or conventional therapy alone (control). Weights are from random-effects model.

**FIGURE 6:**
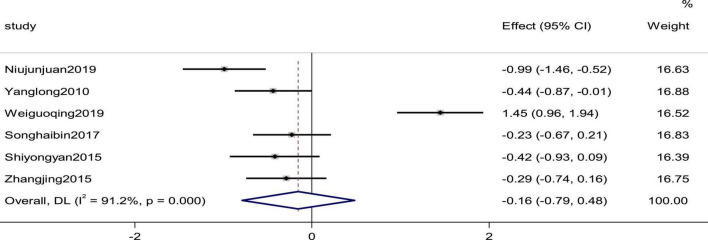
Forest plot of the diastolic pressure in patients with heart failure treated with conventional therapy plus arotinolol (experimental) or conventional therapy alone (control). Weights are from random-effects model.

Six studies reported the systolic pressure and diastolic pressure as outcomes. High heterogeneity (*p*_Systolic pressure_ = 0.000, *I*^2^_Systolic pressure_ = 90.2%; *p*_diastolic pressure_ = 0.000, *I*^2^_diastolic pressure_ = 91.2%) prompted us to use a random effects model to combine the effect sizes. The results showed that the differences between the two groups were not statistically significant (SMD_Systolic pressure_ = −0.09, 95% CI [−0.69, 0.51], *p* = 0.775; SMD_diastolic pressure_ = −0.16,95% C I [−0.79, 0.48], *p* = 0. 632; Figures 5, 6).

### Heart rate

**FIGURE 7:**
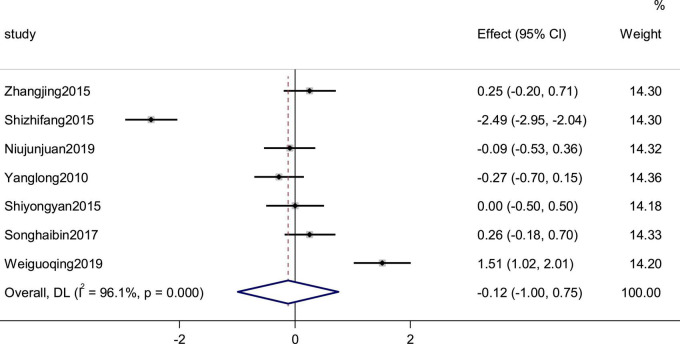
Forest plot of the heart rate in patients with heart failure treated with conventional therapy plus arotinolol (experimental) or conventional therapy alone (control). Weights are from random-effects model.

Seven studies reported the heart rate as an outcome. High heterogeneity (*p* = 0.000, *I*^2^ = 96.1%) prompted us to use a random effects model to combine the effect sizes. The results showed that the data were not statistically significantly different (SMD = −0.12, 95% CI [−1.00, 0.75], *P* = 0.787; Figure 7).

### Cardiac index

**FIGURE 8:**
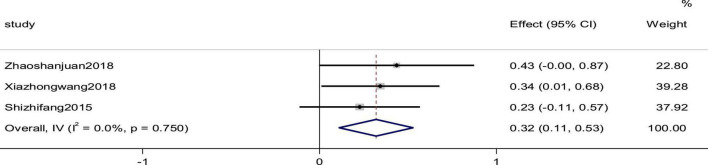
Forest plot of the cardiac index in patients with heart failure treated with conventional therapy plus arotinolol (experimental) or conventional therapy alone (control).

Three studies reported the cardiac index as an outcome. The heterogeneity between studies was small (*p* = 0.750, *I*^2^ = 0), and the results showed that the experimental group was more effective than the control group in improving the efficiency of cardiac index, using a fixed-effects model to combine the effect sizes (SMD = 0.32, 95% CI [0.11, 0.53], *p* = 0.03; Figure 8).

### Stroke volume

**FIGURE 9:**
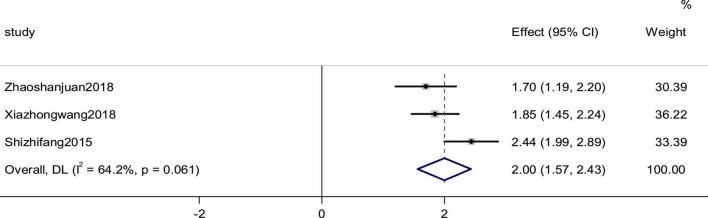
Forest plot of the SV in patients with heart failure treated with conventional therapy plus arotinolol (experimental) or conventional therapy alone (control). Weights are from random-effects model. Weights are from random-effects model.

Three studies reported the SV as an outcome. High heterogeneity (*p* = 0.061, *I*^2^ = 64.2%) prompted us to use a random effects model to analyze the data. The meta-analysis results indicated that a combination of western medicine and arotinolol could further increase SV compared with western medicine alone (SMD = 2.00, 95% CI [1.57, 2.34], *p* = 0.000; Figure 9).

### Brain natriuretic peptide

**FIGURE 10:**
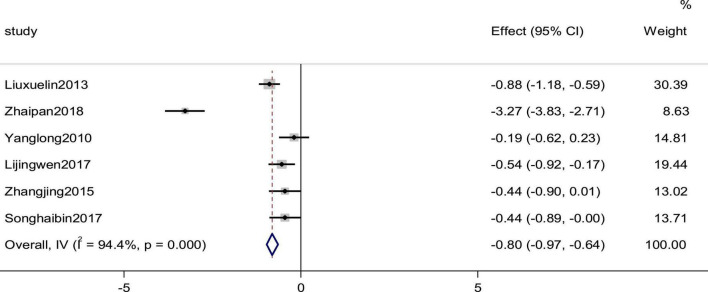
Forest plot of BNP in patients with heart failure treated with conventional therapy plus arotinolol (experimental) or conventional therapy alone (control).

Six studies reported the BNP as an outcome. High heterogeneity (*p* = 0.000, *I*^2^ = 94.4%) prompted us to use a random effects model to analyze the data. The meta-analysis results indicated that a combination of western medicine and arotinolol could further decrease BNP compared with western medicine alone (SMD = −0.804, 95% CI [−0.97, −0.64], *p* = 0.000; Figure 10).

### Hypersensitive C-reactive protein

**FIGURE 11:**
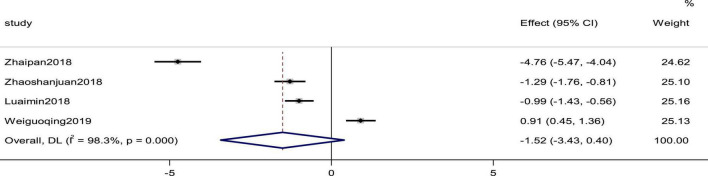
Forest plot of Hs-CRP in patients with heart failure treated with conventional therapy plus arotinolol (experimental) or conventional therapy alone (control). Weights are from random-effects model.

Four studies reported the Hs-CRP as an outcome. The heterogeneity was high (*p* = 0.000, *I*^2^ = 98.3%); therefore, a random effects model was used to combine the effect sizes. The results showed no statistically significant differences in the data (SMD = −1.52, 95%CI [−3.43, 0.40], *p* = 0.121; Figure 11).

### Left ventricular end diastolic volume

**FIGURE 12:**
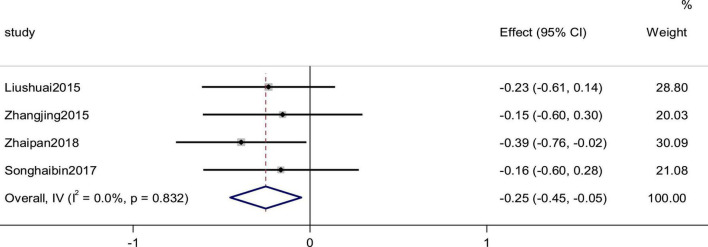
Forest plot of LVEDV in patients with heart failure treated with conventional therapy plus arotinolol (experimental) or conventional therapy alone (control).

Four studies reported the LVEDV as an outcome. The heterogeneity among studies was small (*p* = 0.832, *I*^2^ = 0), and the results showed that the experimental group was more effective than the control group in improving the efficiency of CI using a fixed-effects model to combine the effect sizes (SMD = −0.25, 95% CI [−0.45, −0.05], *p* = 0.015; Figure 12).

### Left ventricular end diastolic diameter

**FIGURE 13:**
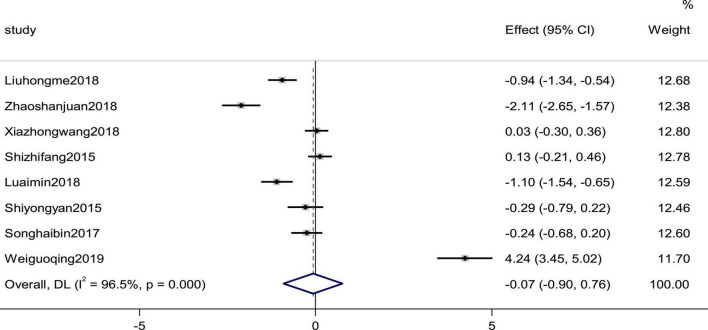
Forest plot of LVEDD in patients with heart failure treated with conventional therapy plus arotinolol (experimental) or conventional therapy alone (control). Weights are from random-effects model.

Eight studies reported the LVEDD as an outcome. There was high heterogeneity (*P* = 0.000, *I*^2^ = 96.5%); therefore, a random effects model was used to combine the effect sizes. The results showed no statistically significant differences in the data (SMD = −0.07, 95% CI [−0.90,0.76], *p* = 0.870; Figure 13).

### Adverse events

A systematic review of 1,717 patients found that the prognosis of patients with CHF was better when treated with conventional therapy plus arotinolol than with CHF therapy alone. This study showed that the combination of conventional therapy with arotinolol further improved LVEF, CI, SV, and reduced BNP and LVEDV. In terms of safety, only four studies with a total sample size of 19 reported adverse events, including nausea, gastric distress, fatigue, and arrhythmias. Overall, the low incidence of adverse events suggests that arotinolol is safe to treat CHF.

## Discussion

CHF is the final destination of most cardiovascular diseases and the leading cause of death, with a high prevalence and mortality rate, and a 5-year survival rate similar to that of malignant tumors (44). The prognosis for patients with CHF is poor, and the early post-discharge period is a period of physical “vulnerability” in which patients might be rehospitalized because of exertion, cold, and other triggers (45). Patients with poor glycemic and lipid control have a higher rate of rehospitalization and a worse prognosis (46, 47).

Evidence-based medicine has shown that beta-blockers significantly reduce cardiovascular mortality, (11, 48, 49) and have become one of the standard medications for CHF (2). Arotinolol is a third-generation beta-blocker that blocks both alpha and beta receptors, and inhibits the activation of the renin-angiotensin-aldosterone system and the sympathetic nervous system. Compared with conventional β-blockers, arotinolol has less effect on the patient’s glucolipid metabolism (50). Considering that most patients with CHF have diabetes and dyslipidemia, this property of arotinolol will bring some benefit to the prognosis of these patients.

In clinical practice worldwide, BNP has been used as a biomarker for disease diagnosis, risk stratification, and prognostic assessment of CHF (51, 52). BNP was found to be positively associated with the incidence of cardiovascular events in patients with chronic CHF (53). A systematic review of 19 studies (54) showed that every 100 ng/L increase in BNP at admission was associated with a 35% increase in the relative risk of all-cause mortality. The results of the present systematic evaluation showed that the clinical treatment of CHF with the addition of arotinolol was more effective than the conventional CHF treatment regimen, in which arotinolol could effectively improve cardiac function and reduce N-terminal BNP precursor levels in patients. This might be related to the ability of arotinolol to control the heart rate, reduce cardiac output, and inhibit the over-activation of the neuroendocrine system, which leads to a significant prolongation of the diastolic phase, thus further improving myocardial energy metabolism (28).

Overall measurement of left heart function is a strong predictor of prognosis in patients with left heart insufficiency and/or CHF (55). During the progression of heart failure disease, acute or chronic myocardial injury leads to the death of some cardiomyocytes, while the remaining surviving myocardium develops cardiomyocyte hypertrophy and interstitial fibrosis as a result of compensatory effects (56, 57). With continued disease progression, the ventricles undergo structural changes, primarily in the left ventricle, including an increase in ventricular volume and hypertrophy of the myocardium. This change is called ventricular remodeling (58). Enlargement of LVEDD is a manifestation of ventricular remodeling, and the results of the present study showed that arotinolol was able to reduce LVEDD in patients with CHF, thereby delaying ventricular remodeling to some extent and improving patient prognosis. Relevant research shows that (28) arotinolol might be effective to improve endothelial function by modulating nitric oxide levels in patients and significantly reducing collagen levels in blood vessels, thereby improving vessel wall remodeling and indirectly improving left ventricular function.

Cardiac systolic function is usually composed of three parts: LVEF, ventricular torsion, and long axis shortening. LVEF simply measures left ventricular short axis function, as reported by Dunlay et al. (59). It is reported that over time, the decrease in LVEF is related to an increase in mortality, while an increase in LVEF is related to improved survival. The results of the present study showed that arotinolol can enhance LVEF in patients with CHF, improve cardiac systolic function, and has a positive impact on the prognosis of patients. Cardiologists also use SV to evaluate cardiac dysfunction in patients with congestive CHF. The definition of stroke volume is the volume of blood pumped out of the left ventricle of the heart during each systolic cardiac contraction. Compared with other commonly used parameters, SV, as a hemodynamic variable, is becoming more popular to evaluate cardiac pump function and organ perfusion, because it is less affected by compensatory mechanisms. The results of the present study showed that the SV of the patients who took arotinolol was stronger than that of those who did not take the drug (60).

## Conclusion

The results of this study demonstrate possible the efficacy and safety of arotinolol in the treatment of CHF. The present study showed that the combination of conventional therapy with arotinolol further improved the LVEF, CI, and SV, and reduced BNP levels and LVEDV. However, there were some shortcomings. For example, although a low incidence of adverse events has been reported in some studies, the safety of arotinolol remains largely unknown, and clinicians and patients should closely monitor the use of this drug during treatment for CHF. Second, the quality of the randomized controlled trials included in this study was generally not high. Only some of the literature mentioned randomized methods, no studies mentioned allocation concealment methods, and no studies mentioned the use of blinding, which introduces some uncertainty into the accuracy of the results. In the future, we look forward to the inclusion of more studies with high quality and large data samples to provide more scientific and reliable data to support for the clinical application of arotinolol to treat CHF.

## Data availability statement

The original contributions presented in this study are included in the article/Supplementary material, further inquiries can be directed to the corresponding author.

## Author contributions

PH conceived the study. PH, QS, YW, AW, HZ, LG, and ZZ developed and implemented the search strategy. PH and ZZ independently screened the titles and abstracts of all retrieved records. PH, QS, and AW performed the data extraction. PH and YW performed the meta-analysis. PH was wrote the drafting of this manuscript. XM reviewed the manuscript to oversaw the conduct of this study. All authors read and approved the final version of the manuscript.
