# Supplementary material for: Effect of arotinolol on chronic heart failure: A systematic review and meta-analysis of randomized controlled trials
Source: Front Cardiovasc Med. 2022 Dec 14;9:1071387. doi: 10.3389/fcvm.2022.1071387 (PMC9795060; doi:10.3389/fcvm.2022.1071387)

Supplementary Material

Supplementary Table 1. Characteristics of the included studies

References	Course of treatment	N (Control)	N (Trial)	Age （control）	Age (trial)	Course of disease (control)	Course of disease (trial)	Dosage	Outcome indicators
Zhouwugang 2008	12W	40	40	65.2 ± 10. 1	62.5 ± 12. 5			20mg/d	2
Liuxuelin2013	6M	95	95	63.2		3-13		10mg/d	1,2,5
Liushuai 2015	24W	52	58	62.3±8.2				30mg/d	1,2,10
Liuhongmei 2018	2M	53	53	67.83±12.24	67.95±12.37	4.13±2.75		10mg/d	1,2,4
Lijingwen 2017	6m	60	55	64. 3 ± 4. 6	63. 2 ± 2. 2			20mg/d	1,5
Zhangjing 2015	6m	38	38	54.9±7.2				30mg/d	2,3,5,6,10
Zhaipan 2018	6m	58	58	55. 30 ± 5. 23	55. 72 ± 5. 96	7. 56 ± 0. 89	7.32 ± 0. 73	30mg/d	1,2,5,7,10
Zhaoshanjuan 2018	3m	41	41	58±7	59±9	5.8±1.5	5.8±1.4	20mg/d	1,2,4,7,8,9
Xiazhongwang 2018	3m	70	70	57.9±2.5	58.4±2.2	4.2±1.2	3.8±1.4	20mg/d	1,2.4,8,9,
Shizhifang 2015	90d	67	67	59.1±1.9	56.7±2.7	3.9±1.3	4.1±1.1	20mg/d	1,2,4, 6,8.9,
Xiede 2018	8w	40	40	58.2±7.3	59.2±6.4	2.6±1.3	3.2± 1.4	20mg/d	1
Luaimin 2018	3m	45	45	60.73±4.21	61.54±4.15			20mg/d	1,2,4,7
Niujunjuan 2019	6m	39	39	55.1 ± 7.2	54.8 ± 6.3			10mg/d	1,3,6
Yanglong 2010	6m	44	45	60 一 80				50mg/d	1,2,3,5,6
Weiguoqing 2019	24m	45	45	60.5±2.5	62.5±3.5			30mg/d	2,3, 4,6,7
Songhaibin 2017	6m	40	40	55. 2 ± 7. 0	54. 8 ± 7. 1			30mg/d	1,2,3,4,5,6,10
Shiyongyan 2015	12W	30	31	45±8	47±8			20mg/d	2,3,4,6,

1.effective rate;2. left ventricular ejection fraction (LVEF);3. blood pressure;4.left ventricular end diastolic diameter (LVEDD);5.brain natriuretic peptide (BNP);6. heart rate;7. hypersensitive C-reactive protein(Hs-CRP);8. cardiac index（CI）;9 stroke volume (SV) ;10 Left ventricular end diastolic volume (LVEDV).

Supplementary Figure 1. Graph showing the risk of bias graph.

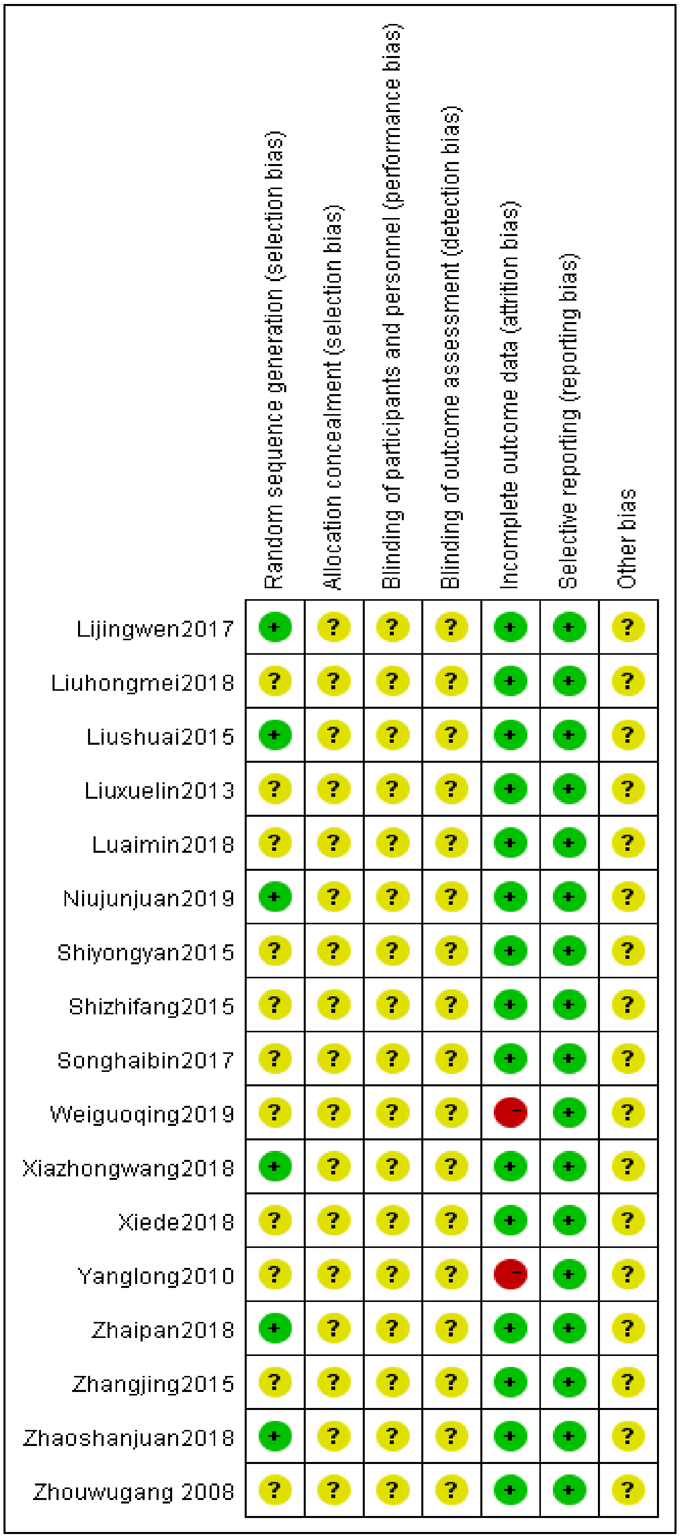

Supplementary Figure 2. Sensitivity analysis of LVEF in patients with heart failure treated with conventional therapy plus arotinolol (experimental) or conventional therapy alone (control).

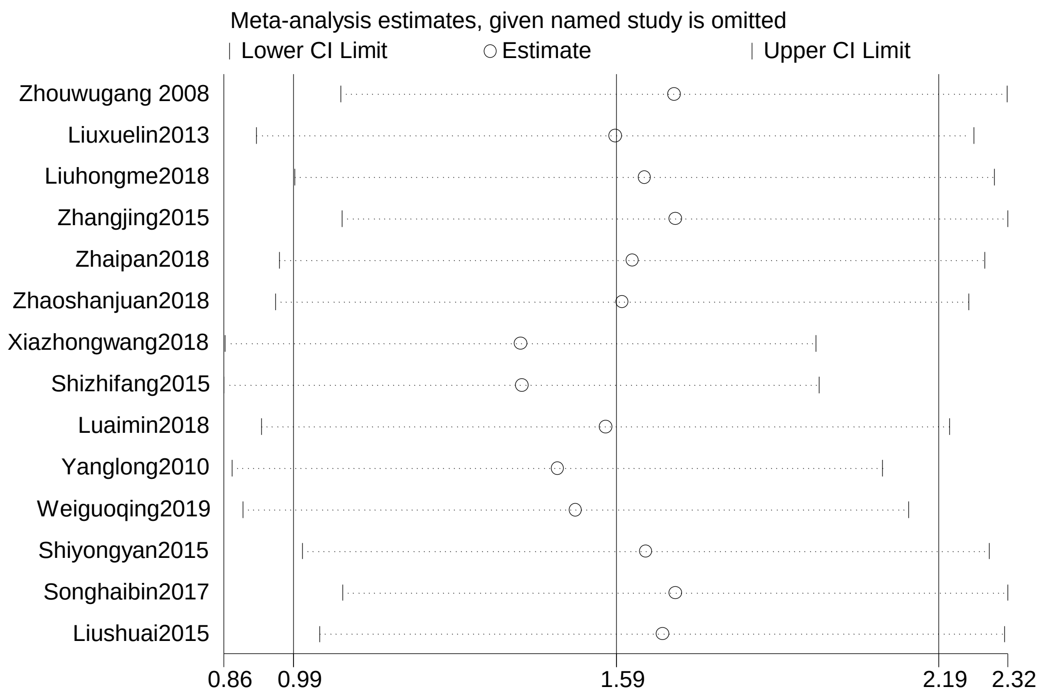

Supplement: Supplementary file 1 [file Data_Sheet_1.pdf]
